# The Little Maid and the Butterfly

**Published:** 1887-11

**Authors:** 


### THE LITTLE MAID AND THE BUTTERFLY.

FROM THE GERMAN OF R. E. WEGENER.

As joyfully tripped a little maid
Within a woodland nigh,
Pausing betimes upon her way,
To gather flowers for a bouquet,
There came a butterfly
Roving‘ dallying as he wist,
And her lips of coral kissed.

Your pardon miss, the rover cried,
My fault prithee excuse,
I only sought for honey sips
Amid the flowers, and so your lips,—
Your ruby lips I chose,
Mistaking them, I freely own,
For roses red and newly blown.

Then spoke the maid in accents kind,
For once you little thing,
I'll pardon freely your offence,
But mark you well from this time hence
When roving on the wing,
These roses that enticed your eye,
Are not for every butterfly.

Translated for Halls Journal of Health.

